# Slice Management for Quality of Service Differentiation in Wireless Network Slicing

**DOI:** 10.3390/s19122745

**Published:** 2019-06-19

**Authors:** Namwon An, Yonggang Kim, Juman Park, Dae-Hoon Kwon, Hyuk Lim

**Affiliations:** 1 School of Electrical Engineering and Computer Science, Gwangju Institute of Science and Technology, Gwangju 61005, Korea; nwan@gist.ac.kr (N.A.); ygkim@gist.ac.kr (Y.K.); 2 Agency for Defense Development, Daejeon 34186, Korea; jmpark12@add.re.kr (J.P.); dhkwon@add.re.kr (D.-H.K.)

**Keywords:** network slicing, isolation, interference, priority, software-defined networking, multiple routing, wireless network

## Abstract

Network slicing is a technology that virtualizes a single infrastructure into multiple logical networks (called slices) where resources or virtualized functions can be flexibly configured by demands of applications to satisfy their quality of service (QoS) requirements. Generally, to provide the guaranteed QoS in applications, resources of slices are isolated. In wired networks, this resource isolation is enabled by allocating dedicated data bandwidths to slices. However, in wireless networks, resource isolation may be challenging because the interference between links affects the actual bandwidths of slices and degrades their QoS. In this paper, we propose a slice management scheme that mitigates the interference imposed on each slice according to their priorities by determining routes of flows with a different routing policy. Traffic flows in the slice with the highest priority are routed into shortest paths. In each lower-priority slice, the routing of traffic flows is conducted while minimizing a weighted summation of interference to other slices. Since higher-priority slices have higher interference weights, they receive lower interference from other slices. As a result, the QoS of slices is differentiated according to their priorities while the interference imposed on slices is reduced. We compared the proposed slice management scheme with a naïve slice management (NSM) method that differentiates QoS among slices by priority queuing. We conducted some simulations and the simulation results show that our proposed management scheme not only differentiates the QoS of slices according to their priorities but also enhances the average throughput and delay performance of slices remarkably compared to that of the NSM method. The simulations were conducted in grid network topologies with 16 and 100 nodes and a random network topology with 200 nodes. Simulation results indicate that the proposed slice management increased the average throughput of slices up to 6%, 13%, and 7% and reduced the average delay of slices up to 14%, 15%, and 11% in comparison with the NSM method.

## 1. Introduction

Network slicing is one of the most important technologies that enable the upcoming fifth generation (5G) mobile networks. In 5G networks, diverse applications with different quality of service (QoS) requirements are required to serve on the same infrastructure at the same time [1]. However, it is highly complicated in general to satisfy the QoS requirements of individual applications such as smartphone, automobile, and massive internet-of-things (IoT) applications on single network infrastructure. Network slicing enables a physical infrastructure to be sliced into logical networks (called slices), which have their own customized topologies and dedicated resources and perform network functions and management policies such as routing, resource scheduling, and admission control [2]. With the network slicing, each type of application can be served on a different slice. For example, one slice can be dedicated to providing reliable and low-latency communications to real-time interactive applications, while another slice is configured for providing high throughput communications for massive IoT applications. Since a slice concentrates on serving the same type of applications with a similar QoS requirement, the configuration or operation of the slice can be tailored to satisfy the QoS requirement. This flexible configuration can be implemented by software-defined networking (SDN), which is a mechanism that separates the control plane from the data plane in a network to manage the data plane flexibly. Using SDN, slices can be constructed as virtual networks on the data plane, and an SDN controller connected to switches on the data plane can manage traffic flows of the slices in a centralized view [3]. Depending on the demands from users, slices can be dedicated to some specific users. These users are called tenants, and multiple slices that serve the same type of applications can be owned by different tenants [4]. Depending on the precedence among tenants or QoS constraints on service layer agreements (SLAs) between the tenants and infrastructure providers, slices may have different priorities. For instance, a higher priority can be given to slices owned by tenants who pay more for premium services or slices with tight and strict QoS constraints on SLAs [5,6].

To enable the slices in providing the guarantee QoS to applications, the resources of each slice need to be isolated. This resource isolation can be achieved by allocating dedicated bandwidths to the slices [7]. In wired networks, the bandwidths are able to be reserved and allocated to slices by managing queues. According to demands from the slices, at each router, dedicated queues of slices are configured and their output rates can be adjusted. However, due to wireless interference between links in wireless networks, resource isolation may be challenging. Due to the omnidirectional signal propagation feature of wireless channels, a transmission of a slice interferes with other transmissions of other slices. This interference not only changes the available actual bandwidths of slices over time but also degrades QoS of the other slices significantly. Therefore, it is most required to mitigate the wireless interference among slices, so that slices established in a wireless network can satisfy QoS requirements of applications successfully. It may be possible to exploit wireless resource management methods that allocate and schedule orthogonal wireless channels, such as frequency or time slot, among links to avoid the interference among the links [8]. However, if the limited number of orthogonal channels is smaller than that of slices, then the interference among the slices cannot be sufficiently avoided.

In this paper, we propose a slice management scheme that mitigates the wireless interference among slices by the prioritized interference-aware routing and admission control. In general, the interference may be more critical to higher-priority slices that are owned by VIP customers, dedicated for QoS-sensitive applications, or used to transmit the emergency data of society. To provide a better QoS to higher-priority slices, we mitigate the interference imposed on each slice according to their priorities by routing flows of each slice with a different routing policy. In the highest-priority slice, traffic flows are routed into shortest paths. In each lower-priority slice, routes of traffic flows are obtained while minimizing the weighted summation of interference imposed on other slices. We assign higher weights to higher-priority slices (i.e., a slice with the highest priority has the highest weight). According to the weights, each slice adjusts the amount of the interference it gives to other slices. Since higher-priority slices receive the lower interference from other slices, a better QoS is provided to applications of the higher-priority slices. Furthermore, to guarantee QoS of slices, we set interference thresholds of slices and maintain the interference imposed on each slice below its interference threshold by the admission control of flows. The main contributions of this work are summarized as follows:

- We propose a joint QoS differentiation method for wireless network slices, which adjusts the interference among slices as well as manages priority queues of slices to differentiate their QoS according to their priorities.
- We propose an interference model between wireless network slices, where an edge-to-edge interference is estimated by the interference power and time portions of slices and the interference among slices is obtained by summing up the edge-to-edge interference.
- We propose inter-slice interfere-aware routing and admission control algorithms for in-band wireless networks, which enable multiple slices to be operated in a single-channel network while minimizing the interference among them.

The remainder of this paper is organized as follows. Section 2 represents related works on the management of wireless network slicing and the interference-aware flow routing. In Section 3, we describe our system model and propose the prioritized slice management schemes with interference-aware routing and admission control mechanisms. Furthermore, simulation results are presented in Section 4. Finally, the conclusion of this paper is presented in Section 5.

## 2. Related Work

In this section, we provide backgrounds and related works with respect to wireless network slicing and interfere-aware routing.

### 2.1. Wireless Network Slicing

Network slicing was originally proposed by 5G working groups as a virtualization technology for multiplexing the future mobile network among diverse applications with different performance and QoS requirements. Several studies have been conducted on network slicing but they mainly focus on slicing wired networks. In wireless networks, since links transmitting data through the same wireless channel interfere with each other, the most important technology for network slicing is to manage radio resources among slices [8].

#### 2.1.1. 5G Radio Access Network Slicing

In the 5G radio access network, several studies have been conducted for the radio resource allocation and scheduling among slices. Ksentini and Nikaein [9] proposed a resource scheduling framework where the mapping between physical resources and user equipment (UE) is investigated by two managers in parallel to mitigate the mapping complexity. A high-level resource manager dedicated for each slice makes a mapping list between UEs and virtual resource blocks (VRBs) and a low-level resource manager schedules VRBs to physical resource blocks. Jiang et al. [10] proposed resource allocation and admission control mechanisms. To evaluate the quality of experiences (QoEs) of users, they normalize data rates of users by maximum bandwidths of slices. Hence, they allocate radio resources to users or slices while maximizing QoEs of users and their admission is controlled by considering the available physical resources and the channel conditions of users. Sallent et al. [11] proposed a multi-layer resource management approach for isolating the radio interference and traffic among tenants in multi-cell radio access networks. In a high-level management layer, the interference among tenants is managed by the inter-cell radio spectrum planning and interference coordination. In a low-level management layer, the traffic among tenants is isolated by the intra-cell packet scheduling and admission control. Ye et al. [12] proposed an SDN-based dynamic bandwidth management framework for two-tier heterogeneous access networks where a macro cell is configured and multiple small cells are connected with each other through the base station (BS) of the macro cell. The proposed mechanism maximizes the resource utilization of the network while satisfying the QoS requirements of slices by enabling a centralized SDN controller to be connected to the BSs of cells via wired links and to optimize the allocation of bandwidth resources among slices in a centralized view. Lin [13] proposed an SDN-based contention-free resource scheduling for 5G wireless networks. The author applied SDN to wireless access networks by simplifying wireless access nodes to remote radio heads with multiple antennas and centralizing their management functions to a baseband server (BBS) pool. The BBS pool schedules wireless resources among flows without the contention based on frequency-division multiplexing resource partitioning, radio beamforming, and power slicing techniques.

#### 2.1.2. Multi-Hop Wireless Network Slicing

Traditionally, in wireless communications, there are resource division methods that enable multiple users to share the same wireless transmission medium by dividing and allocating resources, such as time, space, frequency, and code to users. In wireless mesh networks, a few studies have exploited the resource division methods to configure slices. To achieve the high throughput performance, Lv et al. [14] proposed an orthogonal frequency-division multiple access based wireless network, where different frequency bands (i.e., 2 GHz and 5 GHz) are used for the data transmission and reception at each node. To virtualize a wireless network into multiple slices, they used a generic-algorithm based channel assignment method that schedules subcarriers of frequency bands among virtualized links. Lv et al. [15] also proposed time-division multiple access based wireless network that can be exploited for broadcasting data through multiple paths to guarantee a certain level of packet loss probability. They divided a wireless network into multiple network slices by allocating the different number of time slots into slices according to its data amount and path length. Shrestha et al. [16] proposed a wireless mesh network testbed that can simultaneously accommodate multiple independent experiments based on the frequency–time division multiple access. In the testbed, resources at each node are virtualized by dividing the frequency into orthogonal channels and further dividing each channel into time slots. A slice for running an experiment is configured by allocating and scheduling the frequency and time resources at each node. Figueiredo et al. [17] proposed a virtualization framework for wireless software-defined networks, where the software-defined radio (SDR) and SDN technologies are integrated to slice wireless networks in multiple layers including physical, MAC, and network layers. They designed an architecture of SDR, which enables the radio resources such as frequency, time, and space, to be virtualized and managed by an SDN controller.

### 2.2. Interfere-Aware Routing

Several studies on interfere-aware routing in wireless networks have been conducted, and most of them are based on the combination of fundamental interfere-aware routing metrics. Measurement-based routing metrics provide statistic qualities of links by measuring retransmission count [18], average transmission time [19], and channel busy time [20]. Interference-model-based routing metrics decide whether two links interfere with each other based on the binary interference models [21] or calculate physical interference values from physical interference models [22,23]. Channel-diversity-based routing metrics provide the intra- and inter-flow interference levels based on the diversity of channels used within flows [24]. Ueda et al. [25] proposed a zone-reservation-based QoS routing using directional antennas. They defined an area interfered by a directional antenna that transmits high-priority data as a high-priority zone. A high-priority flow is routed into the shortest path that is maximally disjointed with the high-priority zones, whereas a low-priority flow is routed via a long path to avoid the high-priority zones. Nikseresht et al. [26] proposed a priority-based multi-path routing method for video streams. They classified data packets of a video streaming according to their priorities. High-priority packets are forwarded through the shortest path, while low-priority packets are routed through an interference-disjoint path whose nodes are not within an interference range of paths of high-priority packets. Zhao et al. [27] proposed a cross-layer routing metric for real-time applications. For a path, the packet priority-oriented (PPO) is a summation of priority values of flows passing each node and the packet priority-oriented QoS (PP-QoS) is a weighted summation of the PPO and interfere-aware routing metric values that measured channel busy levels of nodes. Flows are routed through a path with the minimum PPO or PP-QoS.

Unlike the existing works, we focus on the slice management for the in-band wireless network without wireless resource partition, which enables multiple slices to be operated on a single-channel wireless network. In addition, due to the fact that in-band wireless slicing is significantly affected by the interference from the transmissions on other slices, the inter-slice interference among slices is mitigated by interference-aware routing, which, to the best of our knowledge, has not been considered in the literature.

## 3. Prioritized Slice Management

### 3.1. System Model

**Figure 1:**
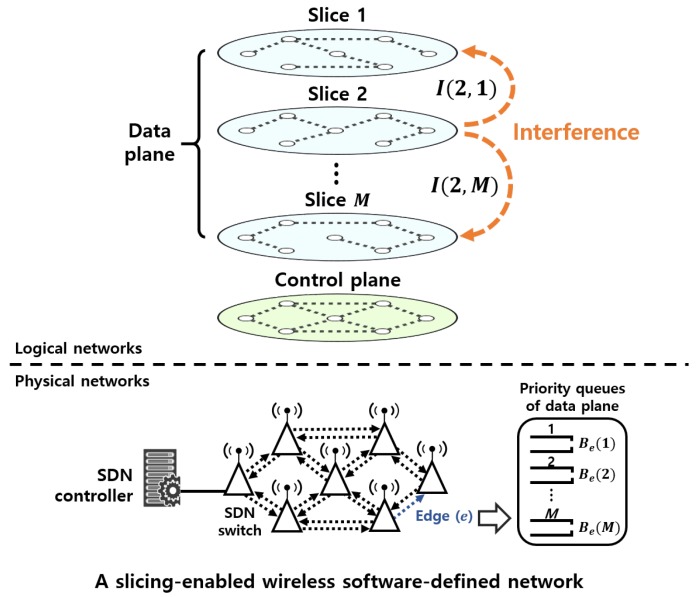
A system model of slicing-enabled wireless software-defined networks.

In this section, we consider a slicing-enabled wireless software-defined network, as shown in Figure 1, in which the control and data planes are separated using different frequency channels and an SDN controller manages the data plane through the control plane. The SDN-enabled switches have two wireless network interfaces used to configure the control and data planes, respectively. The data plane is virtualized into multiple slices, and routing paths of flows in the slices are determined by the SDN controller. Let *M* denote the number of slices operating on the data plane. It is assumed that slice 1 has the highest priority, while slice *M* has the lowest priority. On the data plane, multiple slices serve different priority traffic. The priority of slices is determined by various ways in terms of the traffic flow type such as multimedia traffic and elastic traffic, SLAs between a service provider and a client, and the amount of traffic volume of the slices. For example, the traffic volume of a specific slice is higher than others, the highest priority can be given to the slice for enhancing the QoS of the slice.

In Figure 1, the data plane of the wireless network is represented by a directed graph G(N,E), where N is a set of all wireless nodes and E is a set of directed edges between the nodes. Let F denote a set of all flows. Each slice s∈{1,2,⋯,M} can be expressed as a subgraph $G_{s}\left(N_{s},E_{s}\right)\subseteq G$ and a set of flows belonging to the slice is denoted by $F_{s}$. Each node has the edges to neighbor nodes, which are configured with *M* dedicated priority queues for establishing multiple slices on the wireless network. Routing in slice *s* is represented by a $|F_{s}\left|\times \right|E_{s}|$ routing matrix:

(1) $$R_{s}={\left\{a_{f,e}\right\}}_{|F_{s}\left|\times \right|E_{s}|},$$

where $f\in F_{s}$, $e\in E_{s}$, and $a_{f,e}$ is 1 if edge *e* is used by flow *f* and is 0 if otherwise. To prevent slices with high data rates from occupying the bandwidth of an edge, we set the bandwidths of slices at each edge. Let $B_{e}\left(s\right)$ denote the bandwidth allocated to slice *s* at edge *e*. The transmission rate of slice *s* at edge *e* is bounded by $B_{e}\left(s\right)$. For instance, in Figure 1, the transmission rate of slice 1 at edge *e* cannot exceed its bandwidth $B_{e}\left(1\right)$. The summation of the assigned bandwidths of all slices at each edge satisfies the following inequality:

(2) $$\sum_{s=1}^{M}B_{e}\left(s\right)\leq C_{e}\;\mathrm{for}\;\forall e\in E,$$

where $C_{e}$ is the maximum capacity of edge *e* and $B_{e}\left(s\right)$ is zero if $e\notin E_{s}$. The rate of slice *s* at edge *e* is expressed as

(3) $$\lambda_{e}\left(s\right)=\sum_{f\in F_{s}}a_{f,e}r_{f},$$

where $r_{f}$ is the rate of flow *f*.

Since the data plane of the network uses a single wireless channel, the concurrent transmissions of edges on a slide cause an interference between the edges belonging to the other slides as well as those on the same slide. The aggregate interference that edge $e\in E_{s}$ imposes on edge $e^{\prime }\in E_{s^{\prime }}$ is given by

(4) $$\begin{array}{c} i_{s,s^{\prime }}\left(e,e^{\prime }\right)=\left\{\begin{array}{cc} 0 & \mathrm{if}\;e=e^{\prime }\\ p_{e\to e^{\prime }}\cdot \tau_{e}\left(s\right)\cdot \tau_{e^{\prime }}\left(s^{\prime }\right) & \mathrm{otherwise}, \end{array}\right. \end{array}$$

where $p_{e\to e}$ is the physical interference power that edge $e^{\prime }$ receives from edge *e* and $\tau_{e}\left(s\right)$ is an active time portion for which slice *s* actually transmits data through edge *e* for a unit time. Here, the active time portion is given as

(5) $$\tau_{e}\left(s\right)=\frac{\lambda_{e}\left(s\right)}{C_{e}}.$$

It is worth noting that the interference between two edges is calculated by the interference power and active time portions of the edges. Specifically, the interference occurs at a receiver node when a transmitter node on a slice transmits a signal. However, we simplify the interference modeling between the transmitters and the receivers into the edges on the graph G(N,E). To calculate the interference between two edges, the probability that the transmission of slices collides with each other has to be considered. Due to the fact that the probability that an edge is active corresponds to the active time portion of the edge in Equation (5), the collision probability can be given by the product of active time portions of edges. The larger time portions the slices have, the higher the collision probabilities they have. Therefore, the collision probability of slice *s* and $s^{\prime }$ can be calculated as the product of their active time portions. When routing matrices $R_{s}$ and $R_{s^{\prime }}$ for slices *s* and $s^{\prime }$, respectively, are given, the aggregate interference $I(s,s^{\prime })$ that slice *s* imposes on slice $s^{\prime }$ is given by

(6) $$\begin{array}{c} I\left(s,s^{\prime }\right)=\left\{\begin{array}{cc} \;0 & \mathrm{if}\;s=s^{\prime }\\ \sum_{e\in E_{s}}\sum_{e^{\prime }\in \left\{E_{s^{\prime }}\backslash e\right\}}i_{s,s^{\prime }}\left(e,e^{\prime }\right) & \mathrm{otherwise}. \end{array}\right. \end{array}$$

It is worth noting that the interference $I(s,s^{\prime })$ between the two slices is obtained by summing up the values of edge-to-edge interference in Equation (6). This means that the interference $I(s,s^{\prime })$ is affected by numbers of active edges in the two slices as well as the values of the interference among edges in Equation (4). The large value of $I(s,s^{\prime })$ means that slice *s* imposes the high interference to slice $s^{\prime }$, and the QoS of slice $s^{\prime }$ is largely degraded by the interference.

### 3.2. Prioritized Multiple Routing

Suppose two different slices $s,s^{\prime }\in \left\{1,2,\cdots ,M\right\}$. For each slice *s*, a high interference $I(s,s^{\prime })$ may significantly degrade QoS of other slices. We propose a prioritized routing scheme that reduces the interference imposed on each slice according to their priorities by routing flows of each slice with a different routing policy.

Firstly, we consider a connectivity constraint that all slices have to satisfy. In slice *s*, its routing is determined by routing matrix $R_{s}$. Among the candidates of routing matrix $R_{s}$, a feasible routing has to ensure that the source and the destination of all flows in slice *s* are connected. This connectivity constraint is given by

**Connectivity constraint:**

(7) $$\begin{array}{cc} \sum_{e\in E_{s}}a_{f,e}\cdot \left(o_{e,n}-t_{e,n}\right)= & \left\{\begin{array}{cc} 1 & \;\mathrm{if}\;n={\mathrm{src}}_{f}\\ -1 & \;\mathrm{if}\;n={\mathrm{dst}}_{f}\\ 0 & \;\mathrm{otherwise} \end{array}\right.\\ & \;\;\;\mathrm{for}\;\forall f\in F_{s}\;\mathrm{and}\;\forall n\in N_{s}, \end{array}$$

where ${\mathrm{src}}_{f}$ and ${\mathrm{dst}}_{f}$ are the source and the destination nodes of flow *f*, respectively; $o_{e,n}$ is 1 if edge *e* originates at node *n* and 0 otherwise; and $t_{e,n}$ is 1 if edge *e* terminates at node *n* and 0 otherwise [28]. This constraint ensures that, when the flows go out from the source nodes and come to the destination nodes, they are routed via a single edge only. At intermediate nodes, incoming flow to a node should go out from the intermediate node via an outgoing edge. Secondly, we consider an interference constraint for maintaining the interference imposed on each slice below its interference threshold. A different interference threshold is assigned to slices depending on priorities of slices. Let us denote a threshold the the total interference slice *s* receives from other slices by $\Gamma_{s}$. For slices i,j∈{1,2,⋯,M} and i<j, we set $\Gamma_{i}<\Gamma_{j}$. Then, the interference constraint is given by

**Interference constraint:**

(8) $$\sum_{s=1}^{M}I\left(s,s^{\prime }\right)\leq \Gamma_{s^{\prime }}\;\mathrm{for}\;\forall s^{\prime }\in \left\{1,\cdots ,M\right\}.$$

This constraint means that the aggregated interference slice $s^{\prime }$ receives from other slices *s* (s=1 to *M*) is constrained to be less or equal to its interference threshold $\Gamma_{s^{\prime }}$.

Recall that, from the priorities of slices in Figure 1, slice 1 has the highest priority and slice *M* has the lowest priority. In slice 1, flows are simply routed via the shortest paths. Let $P_{f,s}=\left\{e|a_{f,e}=1\;\mathrm{for}\;\forall e\in E_{s}\right\}$ be a path that includes all edges used to route flow *f* in slice *s*. We find an optimal routing matrix $R_{1}^{*}$ that minimizes the path length $|P_{f,1}|$ of each flow *f* while satisfying the connectivity constraint in Equation (7) and interference constraint in Equation (8).

**Optimization for slice 1:**

(9) $$\begin{array}{cc} R_{1}^{*}= & \arg\min_{R_{1}}\left|P_{f,1}\right|\;\mathrm{for}\;\forall f\in F_{1}\\ & \mathrm{subject}\;\mathrm{to}\;\mathrm{the}\;\mathrm{constraints}\;\mathrm{in}\;\mathrm{Equations}\;(7)\;\mathrm{and}\;(8). \end{array}$$

In slices s=2toM, the flows are routed via the paths that cause minimal interference with other slices. For each slice *s*, we find an optimal routing matrix $R_{s}^{*}$ that minimizes a weighted summation of interference to other slices while satisfying their connectivity constraint in Equation (7) and interference constraint in Equation (8).

**Optimization for slices from 2 to M:**

(10) $$\begin{array}{cc} R_{s}^{*}= & \arg\min_{R_{s}}\sum_{s^{\prime }=1}^{M}w_{s^{\prime }}I\left(s,s^{\prime }\right)\\ & \mathrm{subject}\;\mathrm{to}\;\mathrm{the}\;\mathrm{constraints}\;\mathrm{in}\;\mathrm{Equations}\;(7)\;\mathrm{and}\;(8), \end{array}$$

where $w_{s^{\prime }}$ is an interference weight of slice $s^{\prime }$. For i,j∈{1,2,⋯,M}, we set $w_{i}>w_{j}$ if i<j, i.e., $w_{1}>\cdots >w_{M}$. These interference weights adjust the interference between slices according to their priorities.

**Figure 2:**
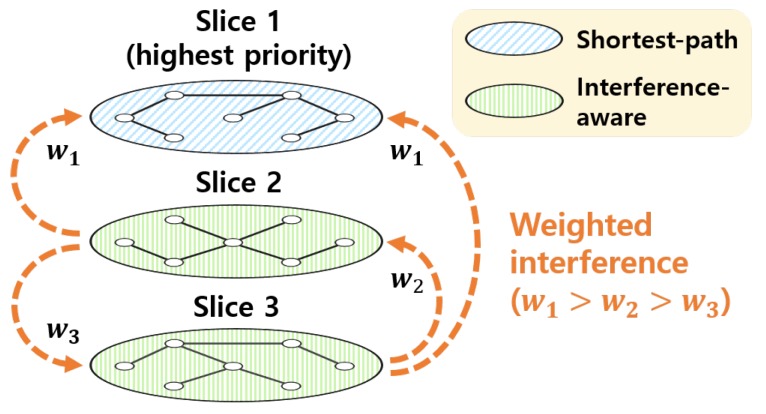
An example of prioritized multiple routing.

For example, suppose there are slices 1, 2, and 3, as shown in Figure 2. They have high, medium, and low priorities. Flows in slice 1 are routed into the shortest paths and flows in slice 2 and 3 can be routed via the paths that cause the minimum interference with other slices. To provide a better QoS to higher-priority slices, the interference imposed on slices is weighted differently depending on the slice priorities. We assign a higher weight to higher-priority slices. In this example, slice 1 has the highest weight $w_{1}$ and slice 3 has the lowest weight $w_{3}$. Based on the weights, flows in slice 2 are routed into paths minimizing a weighted summation of the interference to slices 1 and 3, while routes of flows in slice 3 are determined while minimizing a weighted summation of the interference to slices 1 and 2. As a result, higher-priority slices receive lower interference from other slices. Therefore, the QoS of slices is differentiated by their priorities.

### 3.3. Proposed Algorithms

The optimization for slice 1 in Equation (9) is a well-known shortest-path searching problem that finds a path with the minimum number of hops between the source and the destination nodes, which is known to be solvable in polynomial time. On the contrary, the optimization for slices from 2 to *M* in Equation (10) is a mixed integer nonlinear programming problem, which is generally known as an NP-hard problem [29]. One way of solving the later optimization is by using a brute-force search. However, this method requires a lot of computational time.

We propose a greedy routing method based on the *k*-hop interference models, where two edges within the *k*-hop distance interfere with each other when data are transmitted via the edges at the same time. According to the employed communication protocol, the right value of *k* will be known. In general, 1- and 2-hop interference models are widely used for various types of wireless networks. For instance, k=1 is known to be good for Bluetooth and FH-CDMA networks, while k=2 is appropriate for IEEE 802.11-based networks [30]. Under the *k*-hop interference assumption, the slice interference $I(s,s^{\prime })$ in Equation (6) is simplified to the *k*-hop slice interference as follows:

(11) $$\begin{array}{c} I_{k}\left(s,s^{\prime }\right)=\left\{\begin{array}{cc} \;0 & \mathrm{if}\;s=s^{\prime }\;\\ \sum_{e\in E_{s}}\sum_{e^{\prime }\in \left\{E_{s^{\prime }}\backslash e\right\}}h_{k}\left(e,e^{\prime }\right)\;i_{s,s^{\prime }}\left(e,e^{\prime }\right) & \mathrm{otherwise}, \end{array}\right. \end{array}$$

where $h_{k}\left(e,e^{\prime }\right)$ is a binary function, which is 1 if edge *e* and $e^{\prime }$ are within the *k*-hop distance and 0 otherwise. The interference among edges that are not within the *k*-hop distance is assumed to be negligible and becomes zero by the binary function. In the optimization in Equation (10), let O(s) denote the objective function to be minimized. The objective function under the *k*-hop interference model is given by

(12) $$\begin{array}{cc} O_{k}\left(s\right) & =\sum_{s^{\prime }=1}^{M}w_{s^{\prime }}I_{k}\left(s,s^{\prime }\right)\\ & =\sum_{s^{\prime }=1}^{M}w_{s^{\prime }}\sum_{e\in E_{s}}\sum_{e^{\prime }\in \left\{E_{s^{\prime }}\backslash e\right\}}h_{k}\left(e,e^{\prime }\right)\cdot i_{s,s^{\prime }}\left(e,e^{\prime }\right)\\ & =\sum_{e\in E_{s}}\sum_{s^{\prime }=1}^{M}w_{s^{\prime }}\sum_{e^{\prime }\in \left\{E_{s^{\prime }}\backslash e\right\}}h_{k}\left(e,e^{\prime }\right)\cdot i_{s,s^{\prime }}\left(e,e^{\prime }\right)\\ & =\sum_{e\in E_{s}}A_{s}^{k}\left(e\right), \end{array}$$

where $A_{s}^{k}\left(e\right)$ is defined as an interference value that edge $e\in E_{s}$ imposes on edges of other slices in their *k*-hop area. Then, $A_{s}^{k}\left(e\right)$ is decomposed into a slice rate and an edge cost as follows:

(13) $$\begin{array}{cc} A_{s}^{k}\left(e\right) & =\sum_{s^{\prime }=1}^{M}w_{s^{\prime }}\sum_{e^{\prime }\in \left\{E_{s^{\prime }}\backslash e\right\}}h_{k}\left(e,e^{\prime }\right)\cdot p_{e\to e^{\prime }}\cdot \tau_{e}\left(s\right)\cdot \tau_{e^{\prime }}\left(s^{\prime }\right)\\ & =\tau_{e}\left(s\right)\sum_{s^{\prime }=1}^{M}w_{s^{\prime }}\sum_{e^{\prime }\in \left\{E_{s^{\prime }}\backslash e\right\}}h_{k}\left(e,e^{\prime }\right)\cdot p_{e\to e^{\prime }}\cdot \tau_{e^{\prime }}\left(s^{\prime }\right)\\ & =\frac{\lambda_{e}\left(s\right)}{C_{e}}\sum_{s^{\prime }=1}^{M}w_{s^{\prime }}\sum_{e^{\prime }\in \left\{E_{s^{\prime }}\backslash e\right\}}h_{k}\left(e,e^{\prime }\right)\cdot p_{e\to e^{\prime }}\cdot \tau_{e^{\prime }}\left(s^{\prime }\right)\\ & =\lambda_{e}\left(s\right)\cdot c_{s}^{k}\left(e\right), \end{array}$$

where $c_{s}^{k}\left(e\right)$ is an interference cost of edge $e\in E_{s}$ in its *k*-hop area. The interference cost is given by

(14) $$\begin{array}{c} c_{s}^{k}\left(e\right)=\frac{1}{C_{e}}\sum_{s^{\prime }=1}^{M}w_{s^{\prime }}\sum_{e^{\prime }\in \left\{E_{s^{\prime }}\backslash e\right\}}h_{k}\left(e,e^{\prime }\right)\cdot p_{e\to e^{\prime }}\cdot \tau_{e^{\prime }}\left(s^{\prime }\right), \end{array}$$

which is a deterministic function of edge *e* when routing matrices of other slices are given. Using Equations (12) and (13), $O_{k}\left(s\right)$ in Equation (12) is represented as

(15) $$\begin{array}{cc} O_{k}\left(s\right) & =\sum_{e\in E_{s}}\lambda_{e}\left(s\right)\cdot c_{s}^{k}\left(e\right)\\ & =\sum_{e\in E_{s}}\sum_{f\in F_{s}}a_{f,e}\cdot r_{f}\cdot c_{s}^{k}\left(e\right)\\ & =\sum_{f\in F_{s}}r_{f}\sum_{e\in E_{s}}a_{f,e}\cdot c_{s}^{k}\left(e\right)\\ & =\sum_{f\in F_{s}}r_{f}\sum_{e\in P_{f,s}}c_{s}^{k}\left(e\right)\\ & =\sum_{f\in F_{s}}r_{f}\cdot \sigma_{s}^{k}\left(P_{f,s}\right), \end{array}$$

where $P_{f,s}=\left\{e|a_{f,e}=1\;\mathrm{for}\;\forall e\in E_{s}\right\}$ is a path of flow *f* and $\sigma_{s}^{k}\left(P_{f,s}\right)$ is a cost of path $P_{f,s}$. The path cost is expressed as

(16) $$\begin{array}{c} \sigma_{s}^{k}\left(P_{f,s}\right)=\sum_{e\in P_{f,s}}c_{s}^{k}\left(e\right). \end{array}$$

Under the *k*-hop interference model, the optimization in Equation (10) is simplified as follows:

(17) $$\begin{array}{cc} R_{s}^{k}= & \arg\min_{R_{s}}\sum_{f\in F_{s}}r_{f}\cdot \sigma_{s}^{k}\left(P_{f,s}\right)\\ & \mathrm{subject}\;\mathrm{to}\;\mathrm{the}\;\mathrm{constraints}\;\mathrm{in}\;\mathrm{Equations}\;(7)\;\mathrm{and}\;(8). \end{array}$$

It is worth noting that the value of the objective function is obtained from flow rates (*r*) and path costs ($\sigma_{s}^{k}$). The flow rates are assumed to be constant. If the interference thresholds of slices are much higher than the interference among slices, an optimal solution is given by routing flows into least-cost paths, which is solvable in polynomial time using a shortest-path search in weighted graphs. However, if the traffic amount in the network increases and the interference among slices becomes large, available paths of all flows may have to be investigated to find an optimal solution satisfying the interference constraints of slices. This optimization requires a high computation complexity. Thus, to reduce the complexity, we solve the optimization problem using a greedy method that determines a route of each flow sequentially according to flow rates. Flows with higher rates further increase the value of the objective function when their routing paths become long and $\sigma_{s}^{k}$ is increased. It means that the higher interference is imposed on other slices. Therefore, the high-rate flows can be routed first.

Algorithm 1 shows a greedy routing algorithm. A target slice *s*, a flow set F, and *k* are given as an input. If s=1, the slice is the highest-priority slice, and the flows belonging to the slice 1 are routed using a shortest-path routing algorithm such as Dijkstra and a routing matrix *R* is obtained. On Line 3, the constraints in Equations (7) and (8) are checked. If the constraints are not satisfied, the routing fails and the algorithm is terminated. For slices s=2 to *M*, we route each flow sequentially in decreasing order of flow rates. On Line 7, a routing matrix *R* is initialized. Then, the costs of edges belonging to slice *s* are obtained. On Line 10 and 11, we select a flow with a maximum rate and then find a path minimizing a path cost $\sigma_{s}^{k}$ while satisfying the connectivity and interference constraints. If there is no feasible path, the routing fails and the algorithm is terminated. Otherwise, we update the routing matrix *R* with the path on Line 15. On Line 16, flow *f* is excluded from the flow set F. This process repeats until the flow set F becomes empty. On Lines 19 and 20, the routing matrix of slice *s* is updated with the new one *R* and the flows of slice *s* are routed using the updated routing matrix. Algorithm 1 is executed whenever a new flow is admitted to a slice. In this case, the new flow is added to the flow set F including the existing flows of the slice. It is worth noting that Algorithm 1 also conducts an admission control depending on the constraints in Equations (7) and (8). As, on Lines 4 and 13, if the constraints are not satisfied, the routing fails and a zero matrix is returned. In this case, the admission of the new flow is rejected.

The time complexity of the algorithm can be analyzed using Big O notation. Intuitively, the worst-case time complexity in slice s≥2 is higher than that in slice s=1 because Dijkstra routing algorithm on Line 2 finds only one path for a flow, but the least-cost search on Line 11 may have to find more than one path for each flow if paths do not satisfy the constraints in Equations (7) and (8). Therefore, the worst-case time complexity of the algorithm depends on the case of slice s≥2. Line 8 has the complexity of $O(M|E{|}^{2})$. The loop on Line 9 has the complexity of O(|F|), which is multiplied by the complexity of operations within the loop. Line 11 has the complexity of O(J|N|(|E|+|N|log|N|)) when Yen’s routing algorithm is used to find a path with the minimum $\sigma_{s}^{k}$ among total *J* least-cost paths [31]. The complexity of other lines can be assumed to be executed in a constant time. Then, the time complexity of the algorithm is given as ${O(M|E|}^{2}+J|F||N|(|E|+\left|N|\log|N|)\right)$ by combining the time complexity on Line 8, 9, and 11. This time complexity can be reduced in a real-world computing environment using programming techniques such as dynamic programming.

**Algorithm 1***k*-hop greedy routing. Function greedy-routing (*s*, F, *k*); **Input**: A target slice (*s*), a set of flows (F), number of interference hops (*k*) **Output**: A routing matrix of slice *s*, which is a zero-matrix if the routing fails
 1:**if**s=1**then** 2: R← shortest-path ($G_{1}$, F) 3: **if** *R* does not satisfy the constraints in Equations (7) and (8) **then** 4:  **return** ${\left\{0\right\}}_{\left|F|\times \right|E_{1}|}$ // zero-matrix 5: **end if** 6:**else** 7: R←${\left\{0\right\}}_{\left|F|\times \right|E_{s}|}$ // initialize 8: Calculate $c_{s}^{k}\left(e\right)$ for $\forall e\in E_{s}$ using (14) 9: **while** F≠∅ **do**10:  f← a flow with the highest rate among all flows of F11:  $P_{f,s}\leftarrow$ a path minimizing $\sigma_{s}^{k}$ while satisfying the constraints in Equations (7) and (8) under the given matrix *R*12:  **if** no feasible $P_{f,s}$ **then**13:   **return** ${\left\{0\right\}}_{\left|F|\times \right|E_{s}|}$14:  **end if**15:  Update *R* with $P_{f,s}$16:  F←F−{f}17: **end while**18:**end if**19:$R_{s}\leftarrow R$ // update the routing matrix of slice *s*20:Route flows of slice *s* using the updated matrix $R_{s}$21:**return**$R_{s}$ // the updated routing matrix of slice *s*


## 4. Performance Evaluation

### 4.1. Simulation Environment

We configured the simulation environment for IEEE 802.11-based wireless networks using the NS-2 simulator. Physical and MAC layer parameters were set as shown in Table 1. Nodes were assumed to use the same wireless channel to communicate with each other and they had one wireless interface with a modulation rate of 9 Mb/s. Instead of configuring dedicated queues of slices at each edge, we simply configured the queues at the wireless interface. The queues were DropTail queues, which processed packets in a first-input-first-output manner and dropped packets from the tail when their buffers were full. The queues were scheduled according to their priorities. For *M* slices, we set the bandwidth of each slice at each wireless interface to $\frac{9}{M}$. The maximum output rates of the queues were set to the allocated bandwidths of slices. An input queue for an NS-2 packet was determined by the flow-ID field of the packet. Non-overlapped ranges of flow-ID values were assigned to slices. Flows belonging to a slice had a flow-ID value in the flow-ID range of the slice. According to values of flow-ID field, NS-2 packets were inserted into their service queues. In the optimizations, each slice *s* was associated with a weight of ${\left(\frac{1}{2}\right)}^{s}$, i.e., $w_{1}=\frac{1}{2},\cdots ,w_{M}={\left(\frac{1}{2}\right)}^{M}$. Here, *k* was set to be 2, which is known as an appropriate value to recognize dominant interference links in IEEE 802.11-based networks [30]. Our routing algorithm was implemented in an NS-2 script program. We configured the data plane of SDN using a no ad-hoc routing agent (NOAH) module [32]. In SDN, control packets for managing the network or obtaining routing paths were not transmitted through the data plane. Under the NOAH, wireless nodes did not exchange any control packet with each other in runtime. Instead, the routing rules for each node could be determined in NS-2 script codes. Using this function, our routing algorithm determined the routing paths of traffic flows in the data plane of SDN.

### 4.2. Simulation Scenarios and Results

We performed simulations in a grid topology where the distance between the adjacent nodes was 250 m and a random topology where nodes were deployed randomly in an area of 2.3 square kilometers. We compared the proposed slice management method with a naïve slice management (NSM) method, in which the flows belonging to each slice were routed into shortest paths, and QoS among slices was differentiated by a priority queuing [33]. In the proposed slice management method, routing as well as priority queues of slices was managed to differentiate the QoS of the slices. The flows belonging to each slice were routed into paths that imposed the minimum weighted interference on other slices while satisfying their interference constraints. Here, instead of searching all available paths of flows, we found a maximum 10 paths for each flow in increasing order of path costs and route flows sequentially into least-cost paths satisfying the interference constraints of slices. In Section 4.2.1, we focus on the performance comparison of the *k*-hop greedy routing method in Algorithm 1 with a brute-force search method applied to solve the optimization problem in Equation (10) in a small-sized network with 16 nodes. Then, in Section 4.2.2, we compare the proposed slice management with the NSM in larger-scale networks with 100 and 200 nodes.

#### 4.2.1. Scenario 1: Small-Sized Grid Topology with 16 Nodes

**Figure 3:**
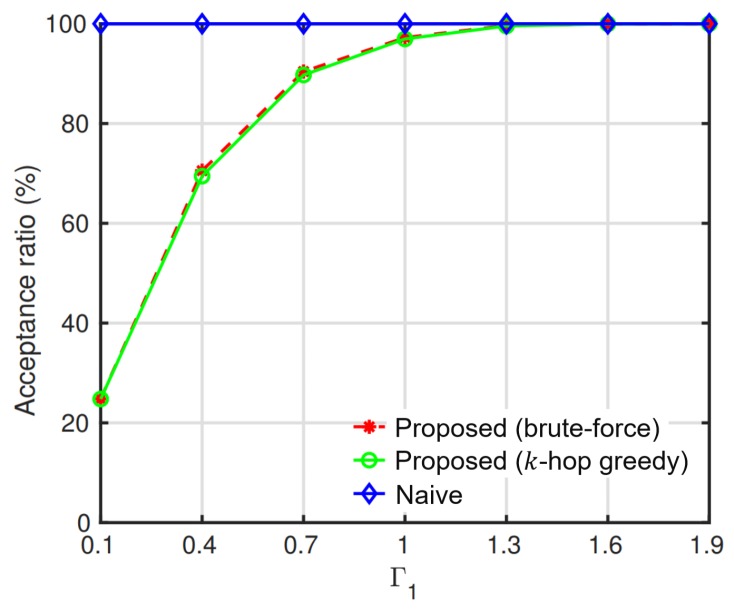
Acceptance ratio of the low-priority slice by the interference threshold of the high-priority slice ($\Gamma_{1}$).

In the proposed slice management, we route flows of each slice using a *k*-hop greedy routing method in Algorithm 1. We compared two cases of the proposed slice management, where the *k*-hop greedy routing and brute-force search methods were applied to route flows, and the NSM where flows were routed with a shortest-path routing method. Due to the computation complexity of the brute-force search method, the performance evaluation was conducted in a small-scale network of 4-by-4 grid topology with small numbers of flows. Two slices with high and low priorities were simulated. Each slice served two flows, and the average rate of flows was 1 Mb/s. Flows of the high-priority slice were routed via shortest paths. Then, we routed flows of the low-priority slice using our *k*-hop greedy routing method, the brute-force search method, and the shortest-path routing method, accordingly. The QoS of slices and the acceptance ratio of the low-priority slice were compared. We repeated the simulation 1000 times and averaged the results. Figure 3 shows the average acceptance ratio of the low-priority flows according to the interference threshold of the high-priority slice ($\Gamma_{1}$). The interference threshold of the low-priority slice ($\Gamma_{2}$) was set to be 2. At each value of $\Gamma_{1}$, the low-priority flows were admitted to the network one by one. When a flow was admitted to the network, it was checked that the interference imposed on the high- and low-priority slices was equal or less than their interference thresholds. If the interference constraints were not satisfied, the flow admission was rejected. In the brute-force search, compared to that of the greedy routing, more low-priority flows were admitted. However, it was observed that the gap of the acceptance ratio between the brute-force search and the proposed greedy routing was small and marginal.

**Figure 4:**
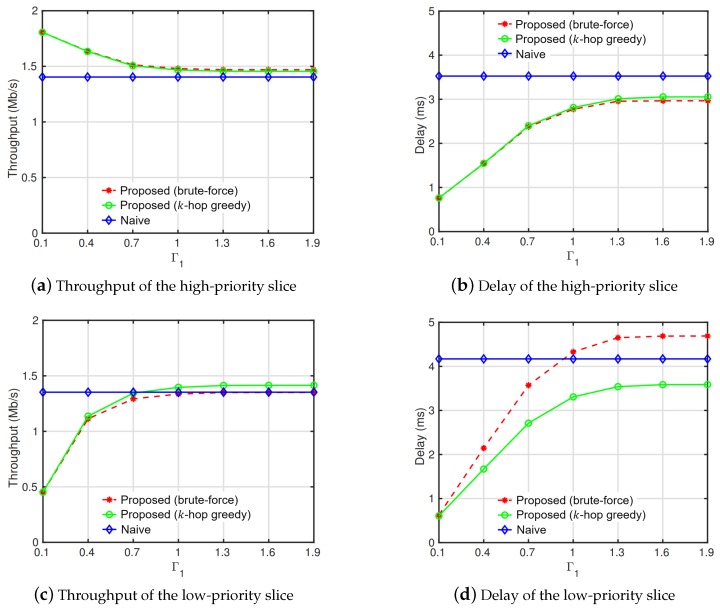
Throughput and delay by the interference threshold of the high-priority slice ($\Gamma_{1}$).

Figure 4 shows the throughput and delay performance of slices. In Figure 4a,b, the throughput and delay of the high-priority slice are presented, respectively. The QoS performance of the brute-force search and greedy routing overwhelmed the shortest-path routing because the interference imposed on the high-priority slice was reduced by routing flows of the low-priority slice into paths causing the minimal interference with the high-priority slice. The brute-force search showed the best QoS performance but the performance gap between the brute-force search and greedy routing was small. Moreover, in the brute-force search, the QoS degradation in the low-priority slice was much higher than the benefits of QoS in the high-priority slice. As shown in Figure 4c,d, the throughput dwindled to lower than the shortest-path routing and the delay largely increased compared to the greedy and shortest-path routing. This was because routing paths of flows in the low-priority slice became tedious to minimize the interference to the high-priority slice. In the greedy routing method, we recognized dominant interference edges that affected the QoS of the high-priority slice based on the *k*-hop interference model and assigned costs to the edges depending on the traffic rate of the high-priority slice within their *k*-hop areas. Based on the costs, flows of the low-priority slice were routed into least-cost paths. Although the QoS of the high-priority slice declined slightly compared to that in the brute-force search in the greedy routing, the QoS of the low-priority slice was highly enhanced. This implies that the performance of our greedy routing outperformed the brute-force search in terms of the average QoS of slices.

#### 4.2.2. Scenario 2: Large-Sized Grid Topology with 100 Nodes

**Figure 5:**
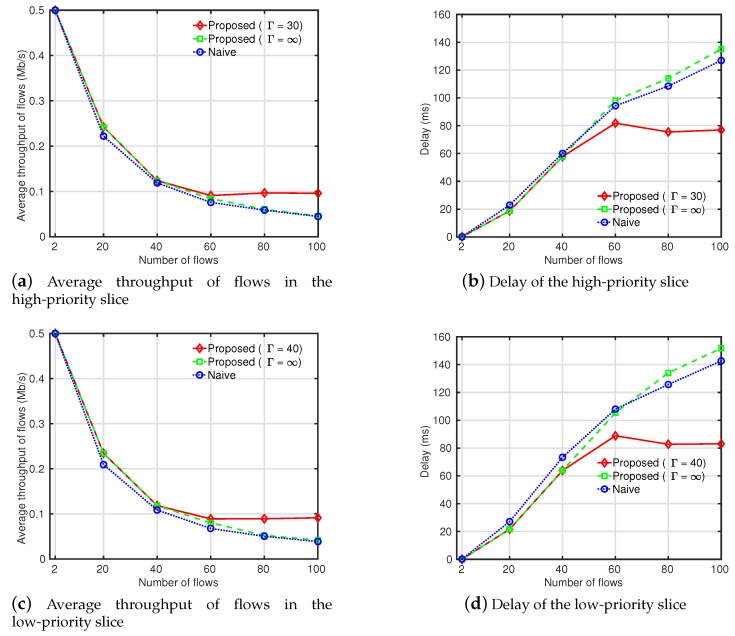
Throughput and delay with respect to different numbers of flows.

We compared the proposed slice management with the NSM with respect to the amount of traffic and numbers of slices in a 10-by-10 grid topology. We set the average rate of flows to 0.5 Mb/s and each flow was admitted to the network randomly one by one. In Figure 5, we compared the proposed slice management methods with the finite gamma (FG), infinite gamma (IFG) and the NSM by the amount of traffic. Two slices with high and low priorities were simulated and their interference thresholds in the proposed slice management with the FG were set to 30 and 40, respectively. Flows were divided into two groups and served by the high- and low-priority slices. Figure 5a,c shows the average throughput of flows in the high- and low-priority slices. As the number of flows increased and the interference among slices became large, the throughput in the proposed slice management with the IFG and the NSM declined. However, the proposed slice management with the IFG showed a higher throughput than the NSM. At 20 flows, the performance gap was highest and dwindled as the amount of traffic increased. Figure 5b,d presents the delays in the high- and low-priority slices. With few flows, the delay in the proposed slice management with the IFG was less than that of the NSM. However, when the network was highly congested, the delay became higher than that of the NSM. As the amount of traffic increased, the interference among the high- and low-priority slices further increased and the probability of packet losses at each edge became higher. Recall that the throughput in the high- and low-priority slices was higher in the proposed slice management with the IFG than in the NSM. This means more packets were successfully transmitted from sources to destinations but they caused more retransmission and queuing delays at each hop. As a result, the end-to-end delays of packets were increased. The QoS degradation caused by network congestion could be avoided using our admission control mechanism. When the number of flows reached 60 in the proposed slice management with the FG, the throughput and delay performance of slices did not decline. This shows that QoS of flows served by each slice could be guaranteed by our admission control mechanism.

**Figure 6:**
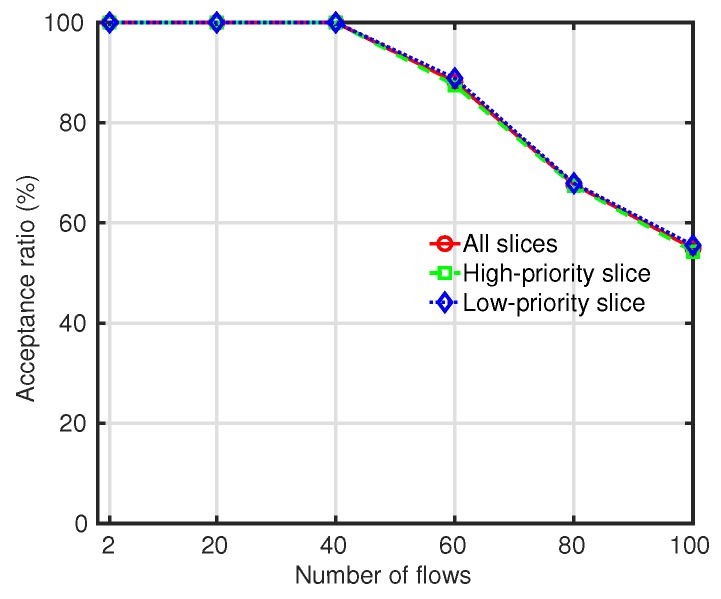
Acceptance ratio with respect to different numbers of flows.

Figure 6 shows the acceptance ratio of slices in the proposed slice management with the FG. Due to the high interference among slices, as the number of flows increased, the acceptance ratio of slices declined. It was observed that the acceptance ratio of the high-priority slice was slightly lower than that of the low-priority slice. In a slice, the admission of a new flow enlarged the traffic amount of the slice and increased not only the interference the slice imposed on other slices but also the interference the slice received from other slices. Since the higher-priority slice had a lower interference threshold, more flows were rejected to satisfy its interference constraint.

**Figure 7:**
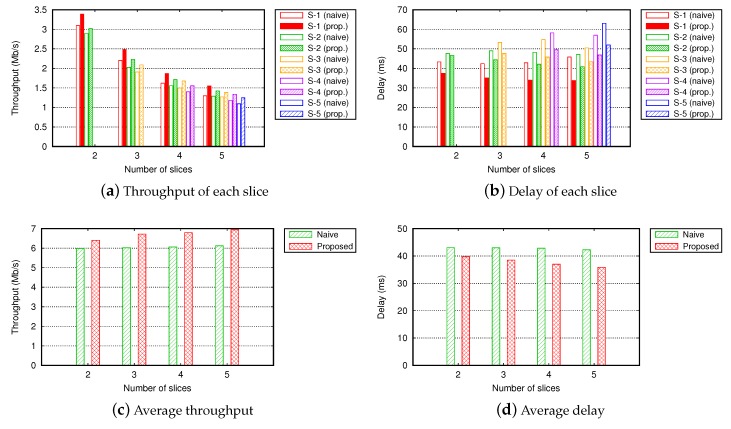
Throughput and delay across different numbers of slices in a grid topology (number of total flows = 30, Γ=∞).

Figure 7 shows the QoS of slices across different numbers of slices. We simulated five slices (S-1 to S-5) with infinite interference thresholds. In total, 30 flows were served in a different number of slices. Figure 7a,b shows the throughput and delay of each slice. In both the proposed slice management and NSM methods, QoS of slices was differentiated according to their priorities. However, in the proposed slice management, the QoS of each slice was higher than that of the NSM by routing flows of each slice into paths causing the minimal interference with other slices. Moreover, the average QoS of slices is worth noting. In Figure 7c,d, the average QoS of all flows in the network was enhanced as the number of slice increased. This was because the granularity for routing the flows became better with more slices. This implies that it is possible to operate many wireless slices with minimal overhead by our proposed slice management scheme.

#### 4.2.3. Scenario 3: Large-Sized Random Topology with 200 Nodes

**Figure 8:**
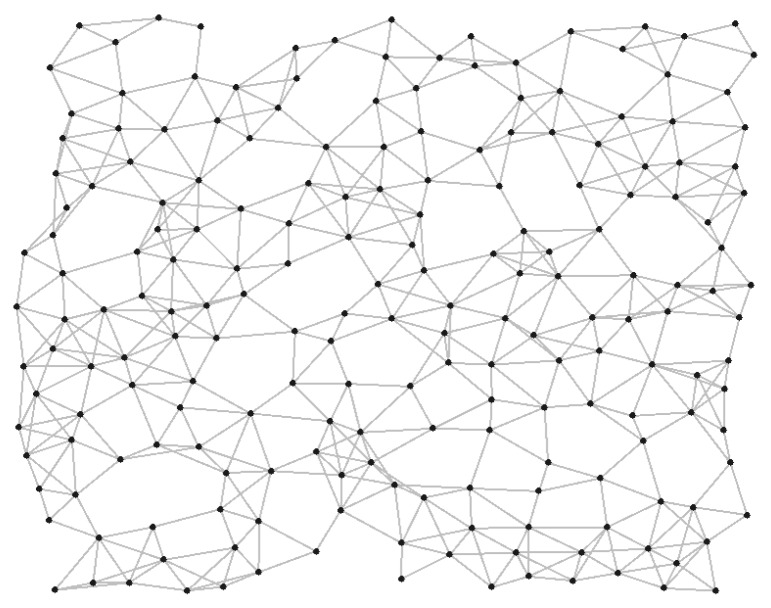
A random topology of 200 nodes.

**Figure 9:**
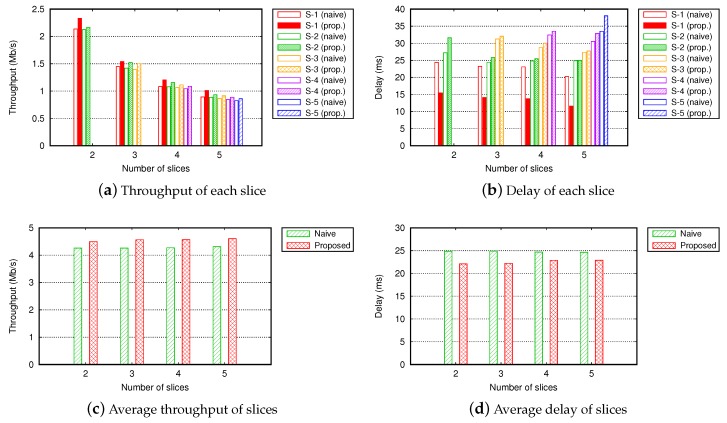
Throughput and delay according to a number of slices in a random topology (number of total flows = 40, Γ=∞).

To evaluate the performance of the proposed slice management in more various network scenarios, we conducted the simulations in a larger-scale random topology shown in Figure 8. In total, 40 flows with an average rate of 0.2 Mb/s were simulated and they were served by different slices with infinite interference thresholds. We compared the QoS of the proposed slice management with the NSM across different numbers of slices. Figure 9a,b shows the throughput and delays of slices that were differentiated according to their priorities. In the proposed slice management, the throughput of each slice was further enhanced compared to the NSM and the delays of high-priority slices were highly reduced. In addition, the average throughput and delay of slices showed a better performance than the NSM, as shown in Figure 9c,d. As the number of slices became large, the performance gap between the proposed slice management and the NSM increased in terms of throughput, while it declined in terms of delay. This was due to a topological characteristic. In a random topology, paths of flows belonging to lower-priority slices tended to be much longer than in a grid topology. As shown in Figure 8, if the higher-priority flows were routed via bottleneck edges, the lower-priority flows were routed into very long paths that bypassed the bottleneck edges to reduce the interference imposed on the higher-priority flows. Therefore, in a random topology, it may be desirable to serve applications that are highly sensitive to delays in the high-priority slice.

## 5. Conclusions

In this paper, we propose a prioritized slice management scheme that differentiates the QoS of wireless network slices according to their priorities. The interference among slices is mitigated by our routing and admission control methods. We propose a greedy routing algorithm that sequentially determines routes of flows belonging to each slice. For each edge, a cost is obtained by summing up the interference the slice imposes on other slices within the *k*-hop area of the edge. We select each flow in decreasing order of its rate and route it into a path with a minimum summation of the costs while satisfying the interference constraints of slices. The admissions of flows are controlled depending on the interference constraints. In the simulation, we evaluated the performance of the proposed slice management method by comparing it with the NSM method, which differentiates QoS of slices using only a priority queuing. The simulation results show that the proposed slice management method not only differentiated QoS of slices according to their priorities but also overwhelmed the NSM method in terms of the throughput and delay performance.

## Figures and Tables

**Table 1:** Simulation parameters of physical and MAC layers in NS-2.

Parameter	Value
Carrier sensing threshold	3.65262 $\times 10^{-10}$ W
Transmission power	0.281838 W
Frequency band	2.4 GHz
Loss factor	1
Minimum contention window	15
Maximum contention window	1023
Slot time	9 μs
Short interframe space	16 μs
Preamble length	144 bits
Length of PLCP header	48 bits
Data rate of PLCP	6 Mb/s
Basic rate	6 Mb/s
Data rate	9 Mb/s
RTS/CTS threshold	1 byte
Limit of short retry	7
Limit of maximum short retry	7
Limit of long retry	4
